# Supplementary material for: Unleashing the potential of noncanonical amino acid biosynthesis to create cells with precision tyrosine sulfation
Source: Nat Commun. 2022 Sep 16;13:5434. doi: 10.1038/s41467-022-33111-4 (PMC9481576; doi:10.1038/s41467-022-33111-4)
Supplement: Supplementary file 1 — Supplementary Information [file 41467_2022_33111_MOESM1_ESM.pdf]

Unleashing the Potential of Noncanonical Amino Acid Biosynthesis to Create Cells with Precision Tyrosine Sulfation

Yuda Chen[1], Shikai Jin[2,3] , Mengxi Zhang[1], Yu Hu[1], Kuan-lin Wu[1], Anna Chung[1], Shichao Wang[1], Zeru Tian[1], Yixian Wang[1], Peter G. Wolynes[1,2,3,4], and Han Xiao[1, 3, 5]*

[1]Department of Chemistry, Rice University, 6100 Main Street, Houston, Texas, 77005
[2]Center for Theoretical Biological Physics, Rice University, Houston, Texas, USA.
[3]Department of Biosciences, Rice University, 6100 Main Street, Houston, Texas, 77005
[4]Department of Physics, Rice University, 6100 Main Street, Houston, Texas, U.S.A.
[5]Department of Bioengineering, Rice University, 6100 Main Street, Houston, Texas, 77005, U.S.A.

* To whom correspondence should be addressed. E-mail: han.xiao@rice.edu;

Table of Contents

Supplemental Figures *1*

Supplementary Figure. 1: Screening reported sulfotransferases with GFP assay. 1

Supplementary Figure. 2: Phylogenetic relationship of all sulfotransferases tested in Fig. 2d 1

Supplementary Figure. 3: Phylogenetic analysis and multiple sequence alignment of *Nn*SULT1C1 and its 9 relatives. 2

Supplementary Figure. 4: Sequence alignment of human cytosolic sulfotransferases (hSULTs) and *Nn*SULT1C1. 3

Supplementary Figure. 5: Superimposition of *Nn*SULT1C1 and 2zvq. 3

Supplementary Figure. 6: Expression condition screening for sfGFP-sTyr production. 4

Supplementary Figure. 7: Kinetics measurment of tyrosine sulfation activity of *Nn*SULT1C1. 4

Supplementary Figure. 8: Flow cytometry analysis of EGFP expression levels of HEK293T and HEK293T-*Nn*SULT1C1. (original data for Fig. 4C) 6

Supplementary Figure. 9: Cellular concentration of sTyr in HEK293T and HEK293T-*Nn*SULT1C1. 6

Supplementary Figure. 10: ESI-MS analysis of EGFP39sTyr from HEK293T cells and HEK293T-*Nn*SULT1C1. 6

Supplementary Figure. 11: SDS-PAGE analysis of thrombin inhibitors purified from LB medium. 7

Supplementary Figure. 12: Protein yields of all thrombin inhibitors used in this study. 7

Supplementary Figure. 13: ESI-MS analysis of all thrombin inhibitors used in this study. a.u. stands for arbitrary unit. 9

Supplementary Figure. 14: Inhibition constants (Ki) of all thrombin inhibitors used in this study 10

Supplementary Figure. 15: Original gel for Fig. 3F. 11

Supplementary Figure. 16: Original gel for Madanin-1. (Fig. 3F left and S11 left) 12

Supplementary Figure. 17: Original gel for Chimadanin. (Fig. 3F right and S11 right) 13

Supplementary Figure. 18: Original gel for Fig. S7. 13

Supplemental Methods *13*
Materials 13
Plasmids Construction 14
Expression and Purification of Proteins. 15
Expression and Fluorescence Measurement of sfGFP 17
E. coli Intracellular sTyr Concentration Measurement 17
Exploration on evolutionary relationship of *Nn*SULT1C1 17
Kinetics Measurement of *Nn*SULT1C1 18
Protein Purification from Mammalian Cells 18
Mammalian Cell sTyr Concentration Measurement 18
Mass Spectra Methods For Proteins 19

Supplemental Figures

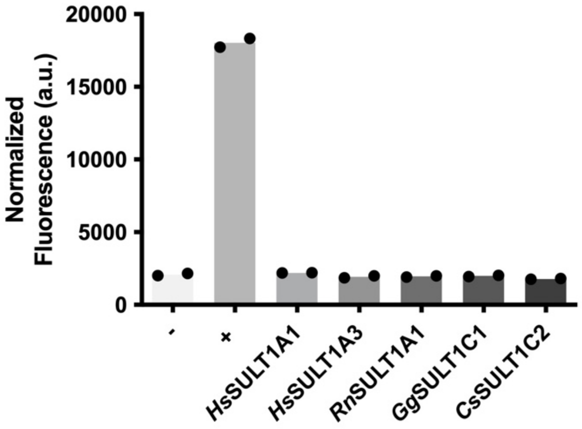

Supplementary Figure. 1: Screening reported sulfotransferases with GFP assay. Data are plotted as means from n=2 independent samples. a.u. stands for arbitrary unit.

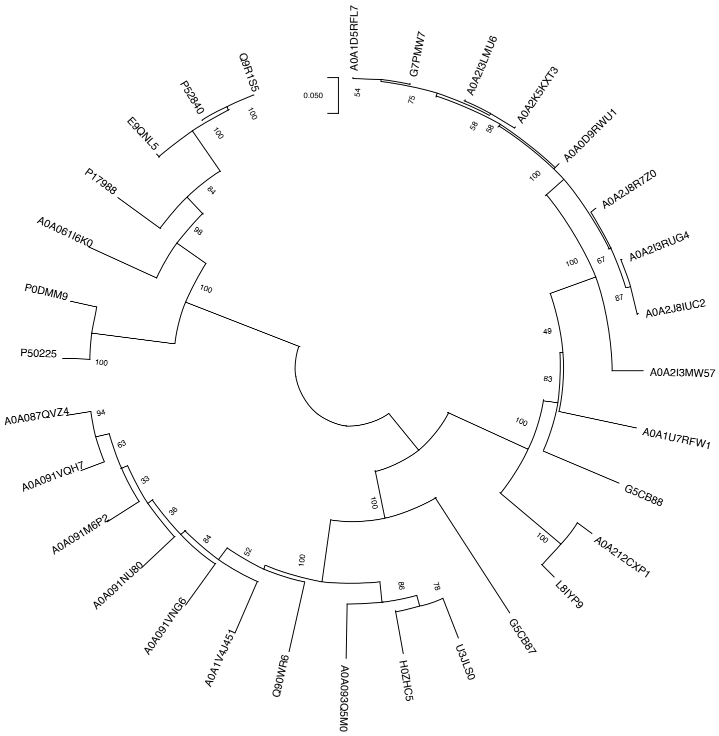

Supplementary Figure. 2: Phylogenetic relationship of all sulfotransferases tested in Fig. 2d. Phylogenetic tree was generated in MEGAX software with UPGMA method. A0A091VQH7 (https://www.uniprot.org/uniprotkb/A0A091VQH7/entry) was named *Nn*SULT1C1 and used for following experiments.

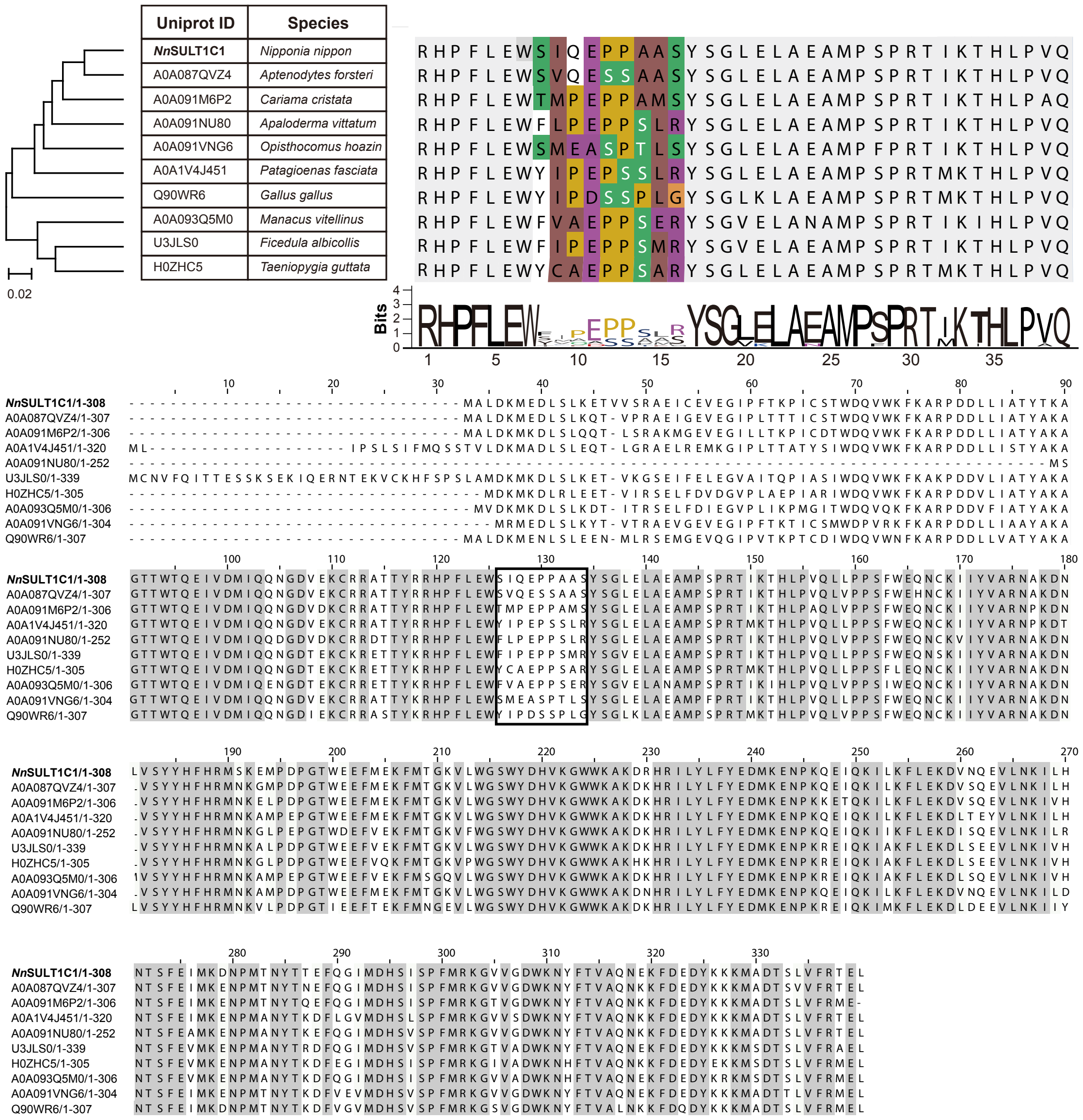

Supplementary Figure. 3: Phylogenetic analysis and multiple sequence alignment of *Nn*SULT1C1 and its 9 relatives. Phylogenetic tree was constructed by UPGMA method in MEGAX and multiple sequences were aligned by ClustalW method.

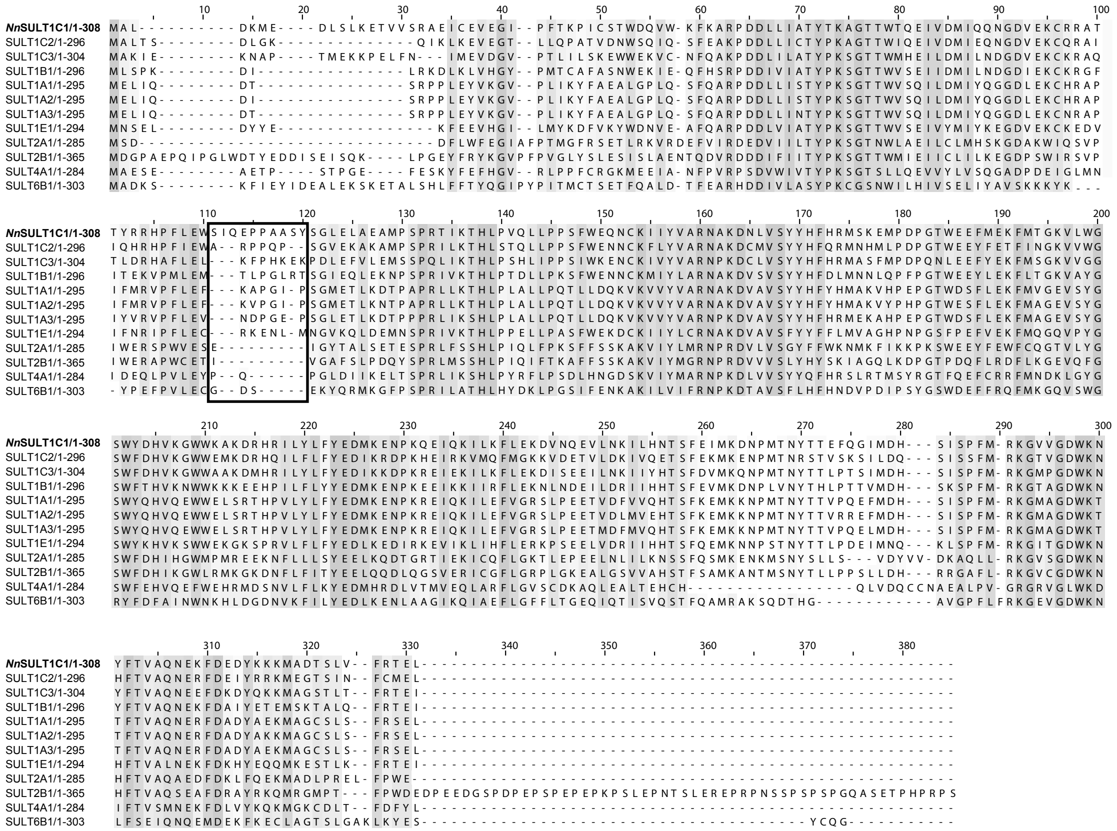

Supplementary Figure. 4: Sequence alignment of human cytosolic sulfotransferases (hSULTs) and *Nn*SULT1C1. The highly variable region we found (SIQEPPAAS) in NnSULT1C1 is aligned well with the reported residues of hSULTs important for substrate recognition.

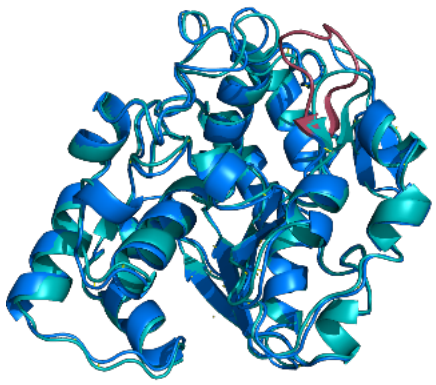

Supplementary Figure. 5: Superimposition of *Nn*SULT1C1 and 2zvq. *Nn*SULT1C1 was shown in blue colour and mouse SULT1D1 (PDB: 2zvq, https://www.rcsb.org/structure/2ZVQ) was shown in cyan colour. The variable region SIQEPPAAS of *Nn*SULT1C1 is shown in red.

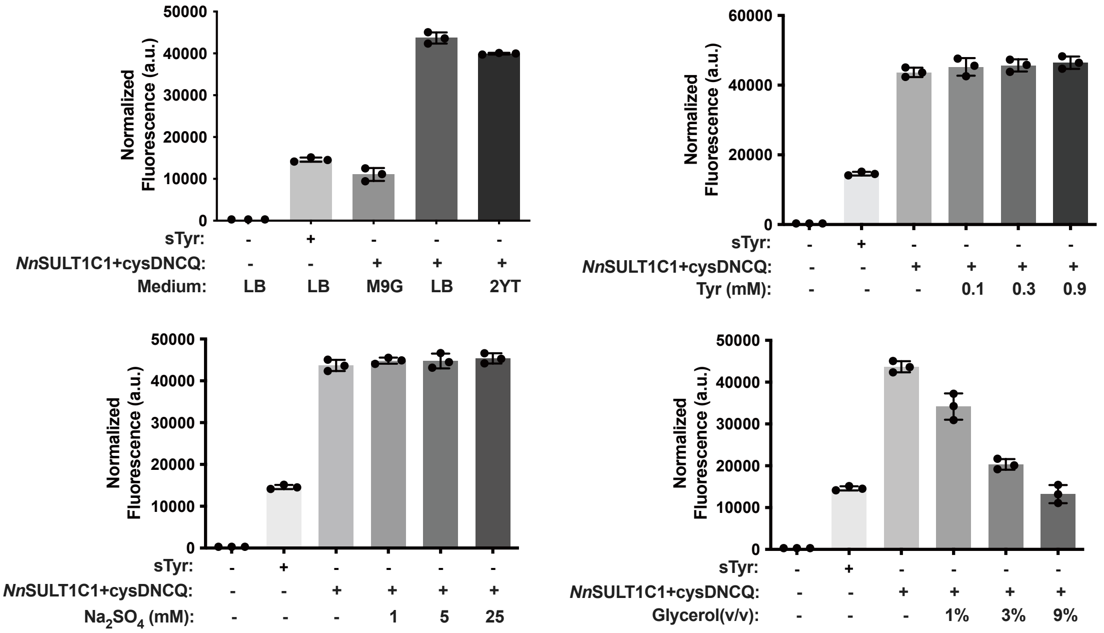

Supplementary Figure. 6: Expression condition screening for sfGFP-sTyr production. The influence of expression medium, tyrosine addition, sulfate addition and glycerol addition on production of sfGFP-sTyr in bacterial cells containing sTyr biosyntheis and genetic incorporation machineries was evaluated with green fluorescent protein assay. Data are plotted as the mean +/- standard deviation from n=3 independent samples. a.u. stands for arbitrary unit.

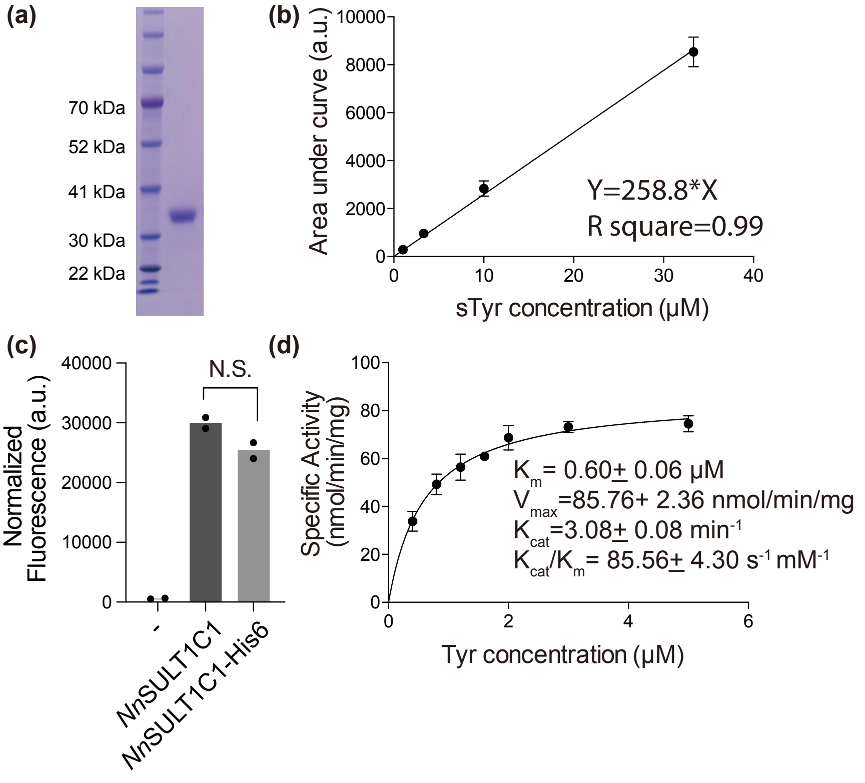

Supplementary Figure. 7: Kinetics measurment of tyrosine sulfation activity of *Nn*SULT1C1. (A) SDS-PAGE analysis of *Nn*SULT1C1-His6 expressed in LB medium. (B) Standard curve of authentic sTyr detected

by SIM mode of ESI-MS. Data are plotted as means of n=2 independent samples. (C) The effect of adding his6 tag to C terminal of *Nn*SULT1C1 on its activity. (D) Kinetics curve of *Nn*SULT1C1 with tyrosine as its substrate. Data are plotted as means of n=3 independent samples. Error bars represent standard deviations from n=3 independent samples. V_{max} and K_m were obtained by fitting the data to Michaelis-Menten equation in Prism. a.u. stands for arbitrary unit.

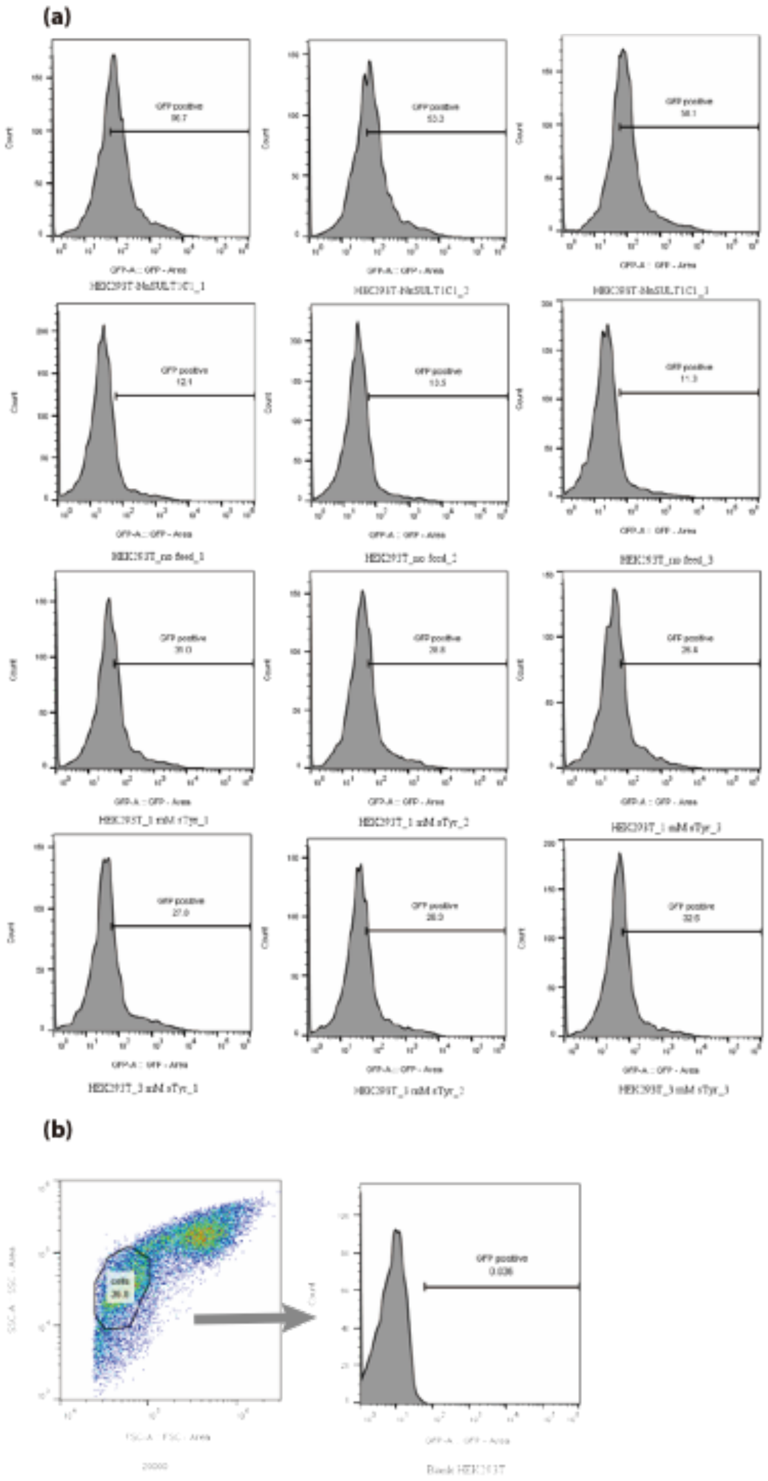

Supplementary Figure. 8: Flow cytometry analysis of EGFP expression levels of HEK293T and HEK293T-*Nn*SULT1C1. (original data for Fig. 4C)

(a) Flow cytometry analysis of EGFP expression in HEK293T and HEK293T-NnSULT1C1 in the presence or absence of external sTyr. There are 3 independent biological replicates for one treatment group, as indicated by the numerical label. (b) Gating strategy used for (a).

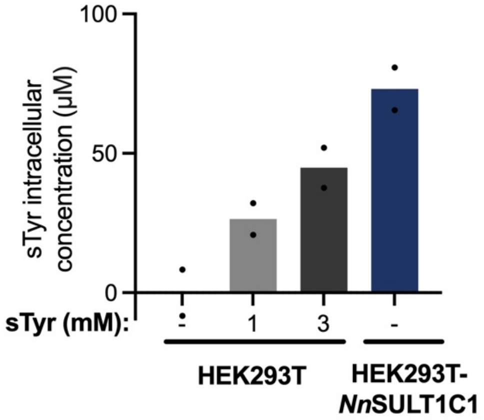

Supplementary Figure. 9: Cellular concentration of sTyr in HEK293T and HEK293T-*Nn*SULT1C1. Indicated concentration of sTyr was added to the culture of HEK293T or HEK293T-*Nn*SULT1C1 for 2 hour. Data are plotted as means from n=2 independent groups.

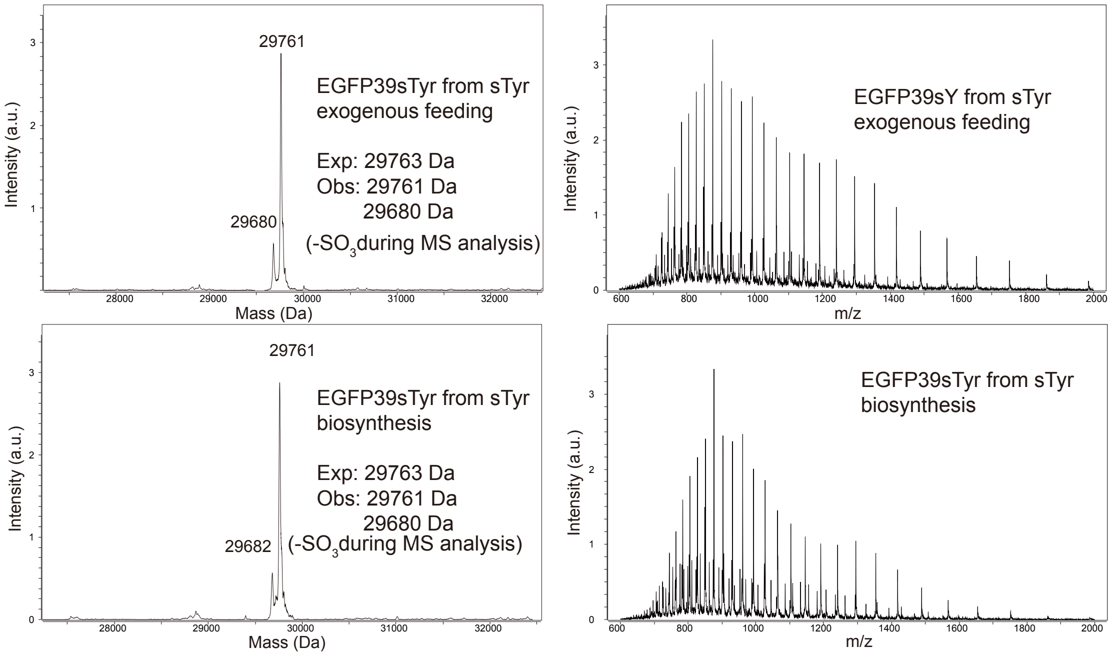

Supplementary Figure. 10: ESI-MS analysis of EGFP39sTyr from HEK293T cells and HEK293T-*Nn*SULT1C1. The expected peak was calculated according to monoisotopic mass of EGFP39sY with N-terminal acetylation. Bottom left spectrum is identical to Fig. 4D. a.u. stands for arbitrary unit.

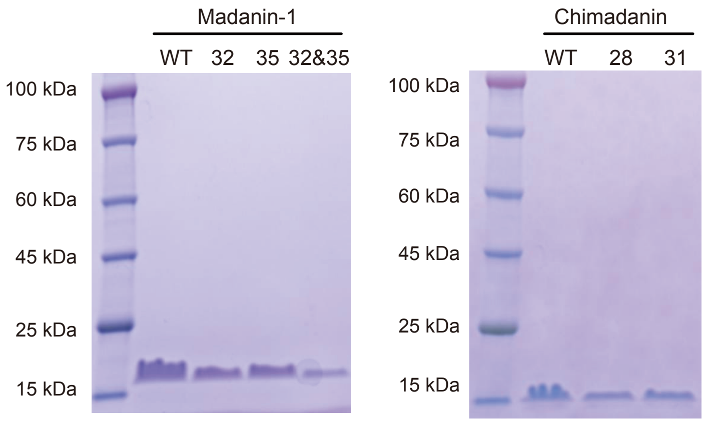

Supplementary Figure. 11: SDS-PAGE analysis of thrombin inhibitors purified from LB medium. sTyr-containing inhibitors are expressed in LB medium with external addition of 3 mM sTyr.

Madanin-1			Chimadanin		
Form	sTyr source	Protein Yield (mg/L)	Form	sTyr source	Protein Yield (mg/L)
Wildtype		3.6	Wildtype		3.0
32sTyr	Biosynthesis	0.3	28sTyr	Biosynthesis	0.11
35sTyr		0.32	31sTyr		0.21
32sTyr35sTyr		0.07	28sTyr31sTyr		0.08
32sTyr	External addition	0.23	28sTyr	External addition	0.26
35sTyr		0.35	31sTyr		0.55
32sTyr35sTyr		0.04	28sTyr31sTyr		0.12

Supplementary Figure. 12: Protein yields of all thrombin inhibitors used in this study.

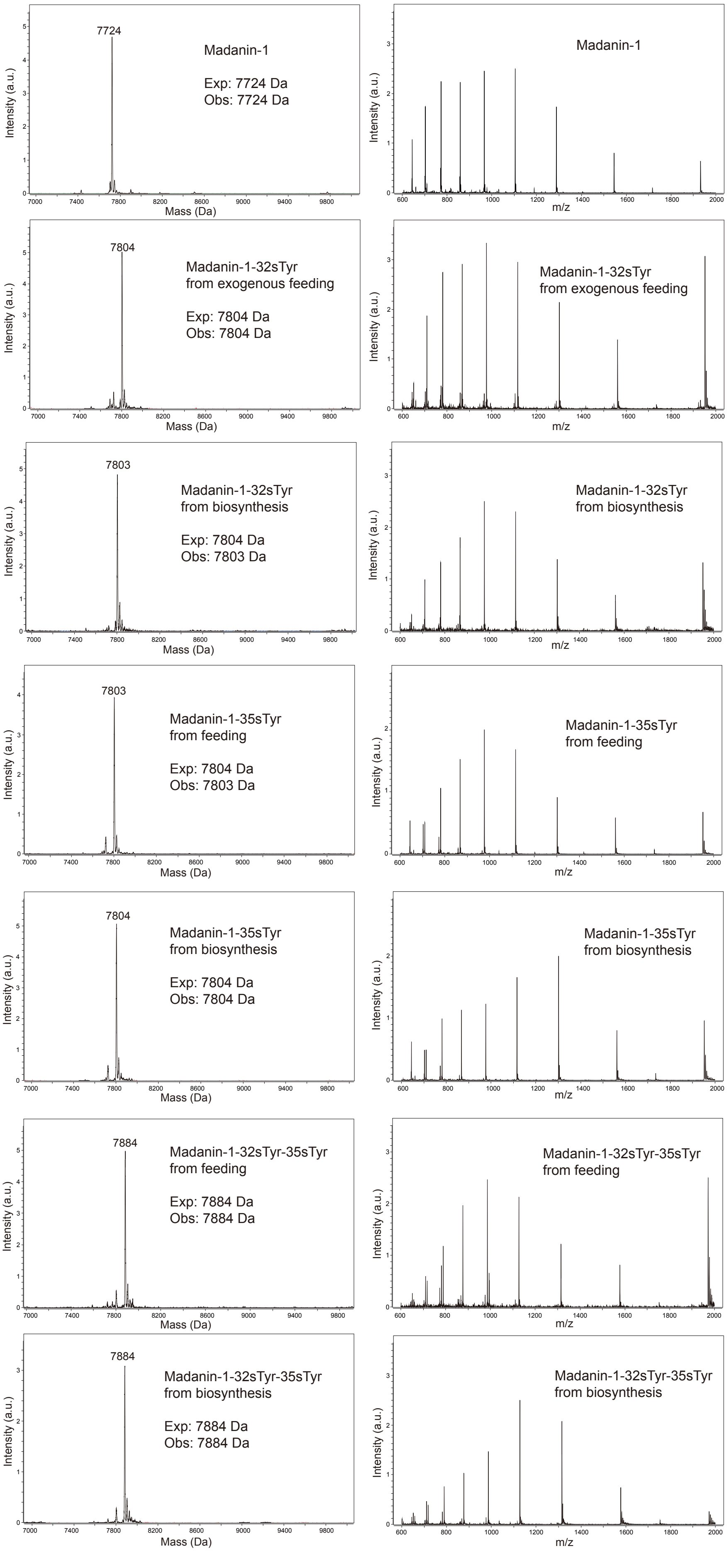

7724
Madanin-1
Exp: 7724 Da
Obs: 7724 Da
Intensity (a.u.)
Mass (Da)
Madanin-1
m/z
7804
Madanin-1-32sTyr
from exogenous feeding
Exp: 7804 Da
Obs: 7804 Da
Madanin-1-32sTyr
from exogenous feeding
7803
Madanin-1-32sTyr
from biosynthesis
Exp: 7804 Da
Obs: 7803 Da
Madanin-1-32sTyr
from biosynthesis
7803
Madanin-1-35sTyr
from feeding
Exp: 7804 Da
Obs: 7803 Da
Madanin-1-35sTyr
from feeding
7804
Madanin-1-35sTyr
from biosynthesis
Exp: 7804 Da
Obs: 7804 Da
Madanin-1-35sTyr
from biosynthesis
7884
Madanin-1-32sTyr-35sTyr
from feeding
Exp: 7884 Da
Obs: 7884 Da
Madanin-1-32sTyr-35sTyr
from feeding
7884
Madanin-1-32sTyr-35sTyr
from biosynthesis
Exp: 7884 Da
Obs: 7884 Da
Madanin-1-32sTyr-35sTyr
from biosynthesis

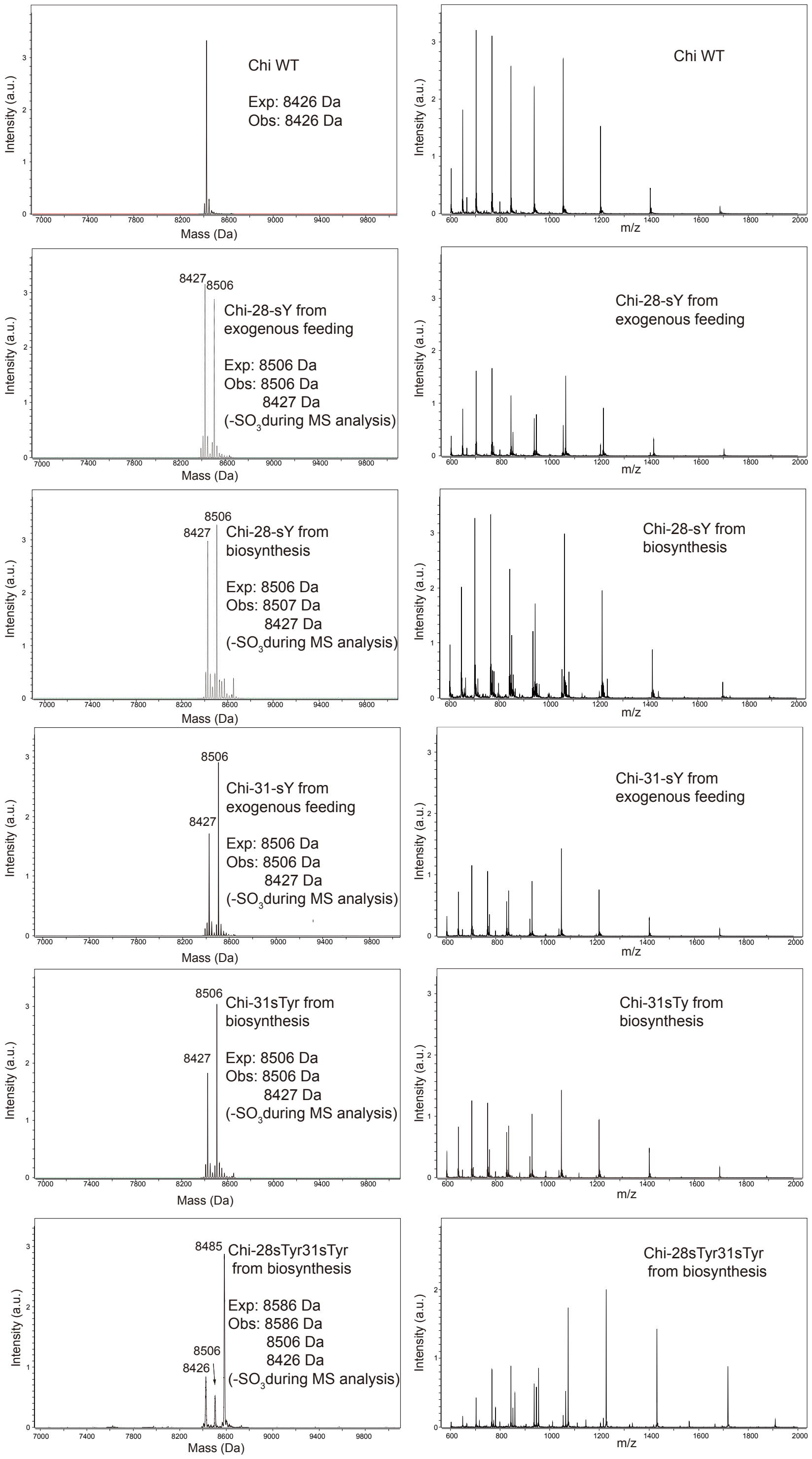

Supplementary Figure. 13: ESI-MS analysis of all thrombin inhibitors used in this study. a.u. stands for arbitrary unit.

Madanin-1			Chimadanin		
Form	sTyr source	K_i (nM)	Form	sTyr source	K_i (nM)
Wildtype		16.0 ± 0.9	Wildtype		12.9 ± 1
32sTyr	Biosynthesis	1.3 ± 0.1	28sTyr	Biosynthesis	0.6 ± 0.1
35sTyr		6.1 ± 0.6	31sTyr		1.5 ± 0.1
32sTyr35sTyr		0.5 ± 0.1	28sTyr31sTyr		0.1 ± 0
32sTyr	External addition	1.8 ± 0.2	28sTyr	External addition	0.5 ± 0.1
35sTyr		6.0 ± 0.3	31sTyr		1.9 ± 0.2
32sTyr35sTyr		0.3 ± 0.1	28sTyr31sTyr		

Supplementary Figure. 14: Inhibition constants (Ki) of all thrombin inhibitors used in this study. K_i ± standard error were calculated based on a tight-binding model, using Morrison equation in Prism. Standard errors were calculated from n=3 independent samples.

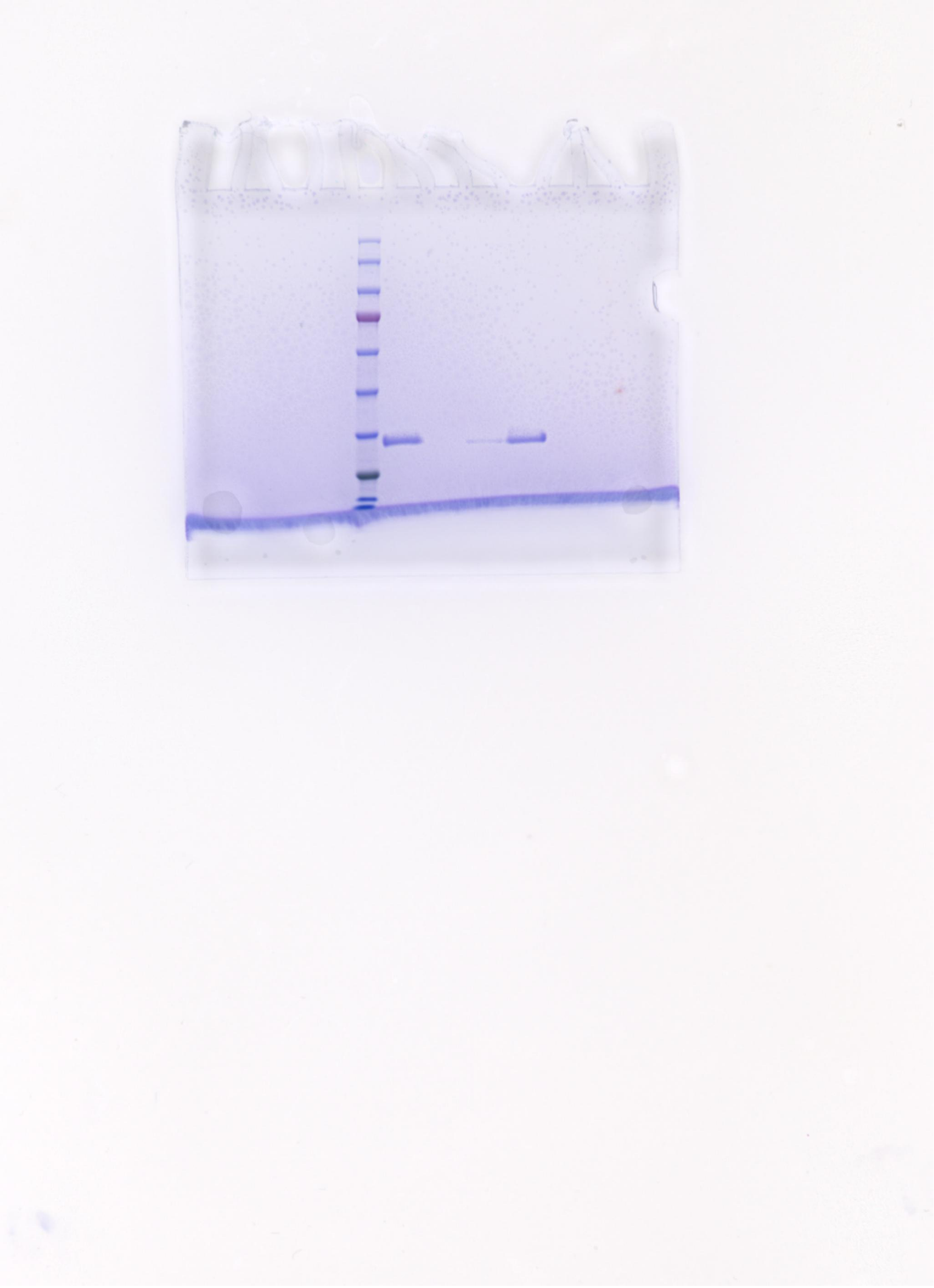

Supplementary Figure. 15: Original gel for Fig. 3F.

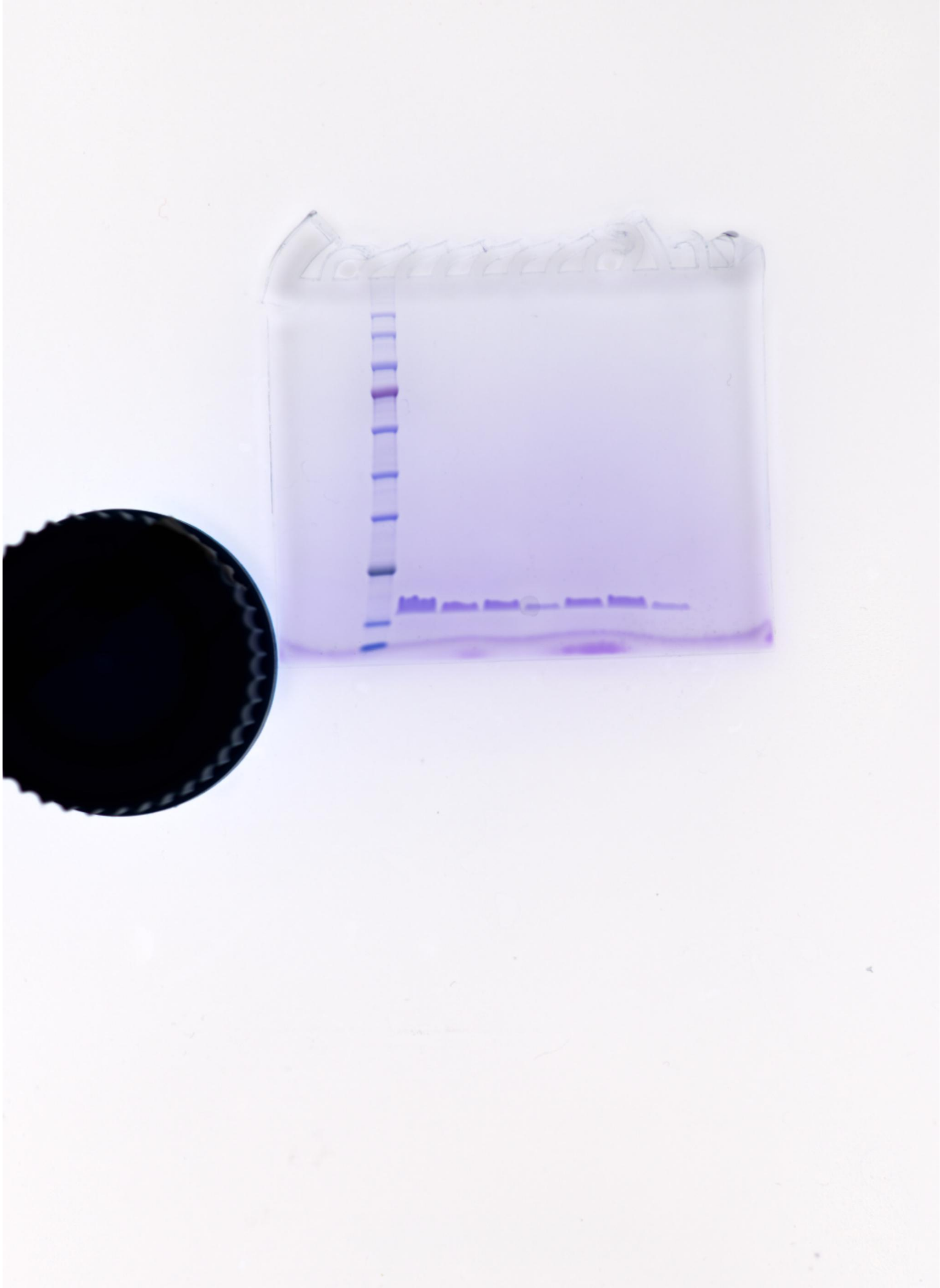

Supplementary Figure. 16: Original gel for Madanin-1. (Fig. 3F left and S11 left)

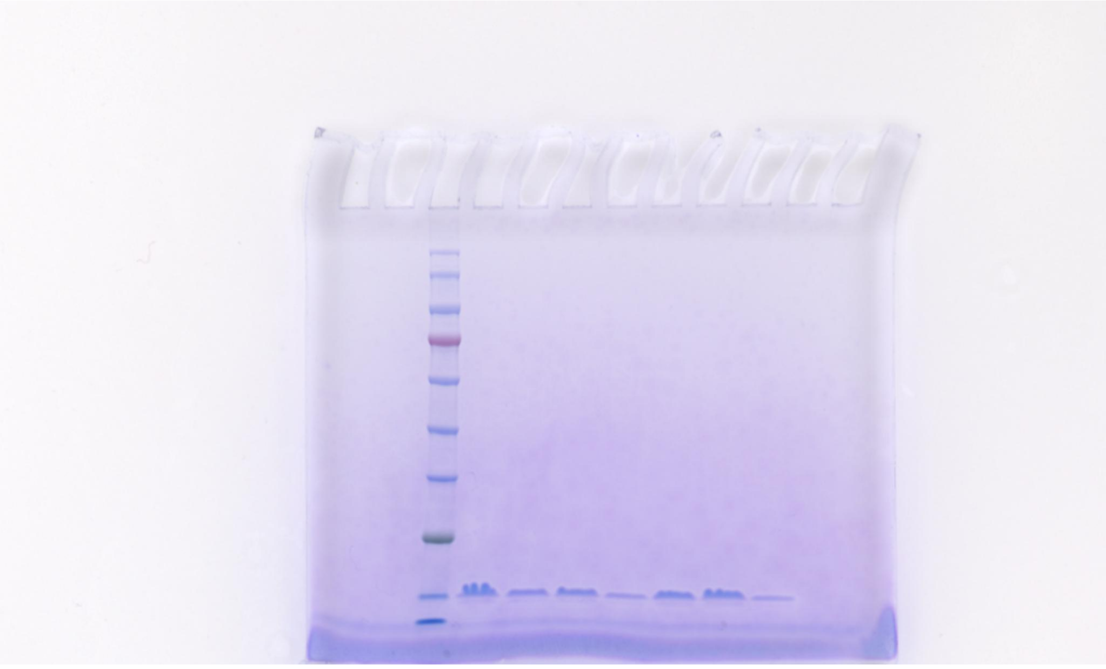

Supplementary Figure. 17: Original gel for Chimadanin. (Fig. 3F right and S11 right)

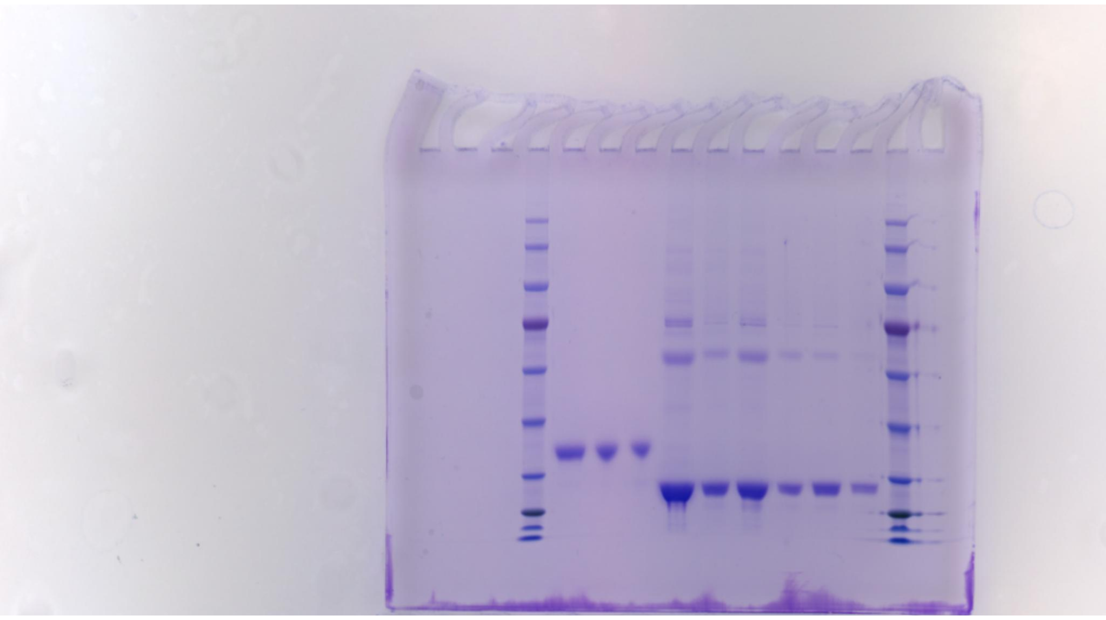

Supplementary Figure. 18: Original gel for Fig. S7.

(The lane next to ladder is *Nn*SULT1C1, proteins in the rest lanes are not relevant to this study)

Supplemental Methods

Materials

LB agar and 2YT were obtained from BD Difco™. Isopropyl-β-D-thiogalactoside (IPTG) was ordered from Anatrace. 4-12% Bis-Tris gels for SDS-PAGE was purchased from Invitrogen. Oligonucleotide primers were purchased from Integrated DNA Technologies and Eurofins Genomics (Supplementary Table S1 lists the oligonucleotides used in this report). Plasmid DNA preparation was carried out with the GenCatch™ Plasmid

DNA Miniprep Kit and GenCatch™ Advanced Gel Extraction Kit. BugBuster™ Protein Extraction Reagent was obtained from Novagen (Cat. 70584). Pierce™ universal nuclease was purchased from Thermo Scientific (Cat. 88700). Ni^{2+}-NTA Agarose was obtained from Qiagen (Cat. 30230). M9-glucose minimal medium contain M9 salt (6.78 g/L Na_2HPO_4, 3 g/L KH_2PO_4, 1 g/L NH_4Cl, 0.5 g/L NaCl), heavy metal solution (1 µg/L $CuSO_4 \cdot 5H_2O$, 4 µg/L $MnCl_2 \cdot 4H_2O$, 4 µg/L $ZnCl_2$, 1.2 µg/L $FeSO_4 \cdot 5H_2O$), 1 mM $MgSO_4$, 0.1 mM $CaCl_2$, 5 µg/mL Thiamine, 300 µM Leucine 4 µM D-Biotin, Glucose (4 g/L).

Unless otherwise mentioned, all solvents and chemicals for synthesis were purchased from Alfa Aesar and Fisher Chemical and used as received without further purification, unless otherwise specified.

Plasmids Construction

pUltra-sTyrRS and pAcBac2.tR4-OMeYRS/GFP* were obtained from addgene. pLei-sfGFP134TAG and pEvol-Mj are generous gifts from Dr. Peter Schultz. Piggybac vector and Piggybac transposase plasmids are kind gifts from Dr. Caleb Bashor. The sequences of all DNA oligos used in this study were shown in supplementary table file.

Sulfotransferase-containing pBad plasmids for initial screening were constructed by Gibson Assembling of pBad vector amplified from pBad-HER2-ScFv with Da343&344 and sulfotransferase fragment amplified from synthetic DNA. pEvol-*Nn*SULT1C1 was acquired by Gibson Assembling of pEvol-Mj vector amplified by Da443&444 and *Nn*SULT1C1 amplified from pBad-*Nn*SULT1C1with Da441&442. To generate pEvol-cysDNC, cysDNC cassette was amplified from *E.coli* genome with Da446 and Da447 and inserted into pEvol vector amplified from pEvol-Mj with Da443&445. To generate pEvol-cysDNCQ, cysDNC cassette and cysQ cassette amplified separately from *E.coli* genome with Da446&448 and Da449&450, respectively, were overlapped and Gibson Assembled into pEvol vector amplified from pEvol-Mj with Da443&445. pEvol-*Nn*SULT1C1-cysDNC(Q) were generated by inserting *Nn*SULT1C1 fragment amplified from pBad-*Nn*SULT1C1 into the vector of pEvol-cysDNC(Q) amplified with Da444&454.

To generate the plasmid for sTyr genetic incorporation in mammalian cells, OMeYRS of pAcBac2.tR4-OMeYRS/GFP* was substituted with sTyr-selective EcTyrRS mutant (L71V, D182G, L186M), which was achieved by Gibson Assembling of synthetic sTyrRS amplified with Da462&463 and pAcBac2.tR4-OMeYRS/GFP* vector digested by Xho1 and Nhe1. To integrate *Nn*SULT1C1 into genome of HEK293T, PB-*Nn*SULT1C1 was constructed by Gibson assembling synthetic *Nn*SULT1C1 amplified by Da655&656 and Piggybac vector digested with BsrG1.

pET22b-T5-chi was constructed by Gibson Assembling of synthetic chimadanin sequence amplified with Da556&557 and pET22b-T5-sfGFP151TAG vector digested with Hind3 and Nde1. pET22b-T5-chi-28TAG and pET22b-T5-chi-31TAG were made according to the protocol on NEBaseChanger with Da664&665 and Da584&585, respectively, using pET22b-T5-chi as their template. pET22b-T5-chi-28TAG31TAG were made by following the same protocol with Da666&Da664 with pET22b-T5-chi-31TAG as a template.

pET22b-T5-mad was constructed by Gibson Assembling of synthetic madanin-2 sequence amplified with Da558&559 and pET22b-T5-sfGFP151TAG vector digested with Hind3 and Nde1. pET22b-T5-mad-32TAG and pET22b-T5-mad-35TAG were made according to the protocol on NEBaseChanger with Da586&587 and

Da661&662 with pET22b-T5-mad as a template. pET22b-T5-mad-32TAG35TAG were made by following the same protocol with Da663&Da661 using pET22b-T5-mad-32TAG as a template.

To explore whether sulfotransferase with similar structure with *Nn*SULT1C1 could catalyze tyrosine sulfation, pEvol-mSULT1D1/hSULT1C2-cysDNCQ was constructed by assembling synthetic sulfotransferases amplified by Da832&833/ Da838&839 with pEvol-*Nn*SULT1C1-cysDNCQ vector amplified with Da802&803.

To test the importance of *Nn*SULT1C1 loop (SIQEPPAAS) and residues (T30, I33, W93, E161) involved in substrate binding, their corresponding mutants was obtained by Gibson Assembling pEvol-*Nn*SULT1C1-cysDNCQ fragments amplified with Da858&859, Da871&872, Da873&874, Da875&876, Da877&878.

To express NnSULT1C1 with his6 tag at C terminal for its kinetics measurement, *Nn*SULT1C1 was amplified from pEvol-*Nn*SULT1C1-cysDNCQ with Da687&688 and Gibson Assembled with pET22b-T5-sfGFP151TAG digested with Hind3 and Nde1, which yields pET22b-T5-*Nn*SULT11-His6.

Expression and Purification of Proteins.

E. coli BL21(DE3) cells, transformed with pUltra-sTyrRS, pLei-sfGFP134TAG, and pBad-Empty/pBad-*Hs*SULT1A1/pBad-*Hs*SULT1A3/pBad-*Rn*SULT1A1/pBad-*Gg*SULT1C1/pBad-*Cs*SULT1C2, were grown in 2YT medium at 37°C. The protein expression was carried out in Luria-Bertani (LB) medium with or without 1 mM sTyr addition. When the OD600 of the cell culture reached 0.6, protein expression was induced by the addition of IPTG and *l*-arabinose to a final concentration of 1 mM and 0.2%, respectively. After growth overnight at 30 °C. Cells were harvested by centrifugation at 4,750 × g for 10 min and used for GFP fluorescence and cell optical density measurements. (**Fig.S1**)

BW25113, ΔtrpE BW25113, ΔtyrA BW25113, ΔackA BW25113, ΔptsH BW25113, ΔcysH BW25113 cells transformed with pUltra-sTyrRS, pET22b-T5-sfGFP151TAG, and pEvol-*Nn*SULT1C1/pEvol-Empty, were grown in 2YT medium at 37°C. The protein expression was carried out in Luria-Bertani (LB) medium with or without 1 mM sTyr addition. When the OD600 of the cell culture reached 0.6, protein expression was induced by the addition of IPTG and *l*-arabinose to a final concentration of 1 mM and 0.2%, respectively. After growth overnight at 30 °C. Cells were harvested by centrifugation at 4,750 × g for 10 min and used for GFP fluorescence and cell optical density measurements. (**Fig. 3B**)

ΔcysH BW25113, transformed with pUltra-sTyrRS, pET22b-T5-sfGFP151TAG, and pEvol-Empty/pEvol-*Nn*SULT1C1/ pEvol- *Nn*SULT1C1-cysDNC/pEvol- *Nn*SULT1C1-cysDNCQ, were grown in 2YT medium at 37°C. The protein expression was carried out in Luria-Bertani (LB) medium with or 1 mM sTyr addition. When the OD600 of the cell culture reached 0.6, protein expression was induced by the addition of IPTG and l-arabinose to a final concentration of 1 mM and 0.2%, respectively. After growth overnight at 30 °C. cells were harvested by centrifugation at 4,750 × g for 10 min and used for GFP fluorescence and cell optical density measurements. (**Fig. 3C**)

ΔcysH BW25113 cells, transformed with pUltra-sTyrRS, pET22b-T5-sfGFP151TAG, and pEvol-Empty/pEvol-*Nn*SULT1C1-cysDNCQ, were grown in 2YT medium at 37°C. The protein expression was carried out in Luria-

Bertani (LB) medium. When the OD600 of the cell culture reached 0.6, *Nn*SULT1C1 expression was induced by indicated concentration of *l*-arabinose and grown at 30°C for 6 h. Then the cells were diluted 5 times to OD 0.6. Expression of reporter sfGFP and sTyrRS were induced with 1 mM IPTG and indicated concentration of sTyr was added at same time. Additional *l*-arabinose was also added to maintain its indicated concentration. After growth at 30 °C for 18 hours, cells were harvested by centrifugation at 4,750 × g for 10 min and used for GFP fluorescence and cell optical density measurements. (**Fig. 3D**) Proteins were purified on Ni-NTA resin (Qiagen) following the manufacturer's instructions. The purified protein was used for SDS-PAGE and ESI-MS analysis. (**Fig. 3F-H**)

ΔcysH BW25113 cells, transformed with pUltra-sTyrRS, pET22b-T5-sfGFP151TAG, and pEvol-Empty/pEvol-*Nn*SULT1C1-cysDNCQ, were grown in 2YT medium at 37°C. The protein expression was carried out in Luria-Bertani (LB) medium. When the OD600 of the cell culture reached 0.6, *Nn*SULT1C1 expression was induced by 15 mg/L *l*-arabinose and grown at 30°C for 6 h. Then the cells were diluted 5 times to OD 0.6. Expression of reporter sfGFP and sTyrRS were induced with 1 mM IPTG and indicated concentration of sTyr was added at same time. Additional *l*-arabinose was also added to maintain its final concentration of 15 mg/L. After growth overnight at 30 °C for 18 hours, cells were harvested by centrifugation at 4,750 × g for 10 min and used for measuring cellular sTyr concentration. (**Fig. 3E**)

To express wildtype thrombin inhibitors, BL21(DE3) cells transformed with either pET22b-T5-chi or pET22b-T5-mad were grown in 2YT medium at 37°C. The protein expression was carried out in LB medium. When the OD600 of the cell culture reached 0.6, protein expression was induced by the addition of 0.4 mM IPTG. After growth overnight at 18 °C for 18 hours, cells were harvested by centrifugation at 4,750 × g for 10 min. Proteins were purified on Ni-NTA resin (Qiagen) following the manufacturer's instructions. The purified protein was used for SDS-PAGE and ESI-MS analysis. (**Fig. 5C**)

To express thrombin inhibitors containing sTyr, ΔcysH BW25113 cells, transformed with pUltra-sTyrRS, pET22b-T5-inhibitor-X-TAG, and pEvol-*Nn*SULT1C1-cysDNCQ, were grown in 2YT medium at 37°C. In the control group, ΔcysH BW25113 cells were transformed with pUltra-sTyrRS, pET22b-T5-inhibitor-X-TAG, and pEvol-Empty. When the OD600 of the cell culture reached 0.6, *Nn*SULT1C1 expression was induced by 15 mg/L concentration of *l*-arabinose and grown at 30°C for 6 h. Then the cells were diluted 5 times to OD 0.6. Expression of inhibitor and sTyrRS were induced with 1 mM IPTG and 3 mM sTyr was added to only control cells at same time. Additional *l*-arabinose was also added to maintain its final concentration of 15 mg/L. After growth overnight at 18 °C for 18 hours, cells were harvested by centrifugation at 4,750 × g for 10 min. Proteins were purified on Ni-NTA resin (Qiagen) following the manufacturer's instructions. The purified protein was used for SDS-PAGE and ESI-MS analysis. (**Fig. 5C and S11**)

To test the importance of the variable loop and residues in binding pockets, ΔcysH BW25113 cells, transformed with pUltra-sTyrRS, pET22b-T5-sfGFP151TAG, and pEvol-*Nn*SULT1C1-cysDNCQ with indicated sequence mutations were grown in 2YT medium at 37°C. To test the tyrosine sulfation activity of the top 3 sulfotransferases with similar structure to *Nn*SULT1C1, ΔcysH BW25113 cells, transformed with pUltra-sTyrRS, pET22b-T5-sfGFP151TAG, and pEvol-X sulfotransferase-cysDNCQ were grown in 2YT medium at 37°C. The protein expression was carried out in LB medium. When the OD600 of the cell culture reached 0.6, sulfotransferase expression was induced by 15 mg/L *l*-arabinose and grown at 30°C for 6 h. Then the cells were diluted 5 times

to OD 0.6. Expression of sfGFP and sTyrRS were induced with 1 mM IPTG. Additional *l*-arabinose was also added to maintain its final concentration of 15 mg/L. After growth overnight at 30 °C for 18 hours, cells were harvested by centrifugation at 4,750 × g for 10 min and used for GFP fluorescence and cell optical density measurements. **(Fig. 2B, C, F)**

To express *Nn*SULT1C1 for kinetics measurment, BL21(DE3) cell transformed with pET22b-T5-*Nn*SULT11-His6 was grown in 2YT medium at 37°C. The protein expression was carried out in Luria-Bertani (LB) medium. When the OD600 of the cell culture reached 0.6, protein expression was induced by the addition of 0.3 mM IPTG. After growth overnight at 30 °C for 18 hours, cells were harvested by centrifugation at 4,750 × g for 10 min. Proteins were purified on Ni-NTA resin (Qiagen) following the manufacturer's instructions. The purified protein was used for SDS-PAGE and kinetic assay. (**Fig. S7**)

Expression and Fluorescence Measurement of sfGFP

After sfGFP expression with the methods described above, 0.5 mL cells were harvested by centrifugation at 4,750 × g for 10 min and then suspended with 0.5 ml PBS (pH 7.4). Fluorescence of cells was measured using excitation/emission wavelengths of 395/509 nm. Optical Density at 600 nm was also obatined. The sfGFP fluorescence/OD600 was used as the normalized fluorescence. The error bars represent the standard deviations of 3 independent protein expression trials.

***E. coli* Intracellular sTyr Concentration Measurement**

Cells were harvested by centrifugation at 4,750 × g for 10 min and washed with PBS 7.4 for three times. The cell pellets were re-suspended in 300 μL of bugbuster lysis buffer : toluene (80: 20) solution and shaken at 30 ℃ for 1 h. The resulting lysate was centrifuged at 21000 g for 30 min at 4 ℃. 200 μl supernatant was transferred to a new tube and re-centrifuged at 21000 g for 2 h. 50 μl supernatant from the top was then analyzed using the LC-MS. An Agilent 1260 Infinity II LC System coupled with Single Quadrupole ESI-MS System was used for analysis of all samples. To measure the sTyr ions, ions detected were set to selected ion monitoring (SIM) mode (262 m/z) to detect positive ions of sTyr. Standards of 1 μM, 25 μM, 50 μM, 100 μM, 200 μM, and 400 μM of authentic sTyr (Bachem) were also prepared and analyzed by the same method. Using LC-MS data, a linear standard curve was generated based on peak areas corresponding to sTyr ions and the concentration of sTyr in standards. The standard curve was then used to calculate the concentrations of sTyr from different cell lysates. Each sample was carried out in n=3 independent groups. The intracellular concentration of sTyr in cells was calculated based on the following equation.

$$[\boldsymbol{sTyr\ intracellular}] = \frac{\boldsymbol{sTyr\ concentration\ in\ lysate\ \times volumn\ of\ lysate}}{\boldsymbol{total\ cell\ numbers\ \times\ E.coli\ cell\ volumn}}$$

Total cell numbers were calculated with the approximate values: 8×10^{8} cells per OD600. 0.6 fL was used as an average *E. coli* cell volume.

Exploration on evolutionary relationship of *Nn*SULT1C1

The rooted phylogenetic tree was inferred using the UPGMA method in MEGA X software. The UPGMA algorithm constructs the tree that reflects the genetic distance between protein sequences present in a pairwise similarity matrix. The tree is drawn to scale, with branch lengths in the same units as those of the evolutionary distances used to infer the phylogenetic tree. The evolutionary distances were computed using the Poisson

correction method and are in the units of the number of amino acid substitutions per site. 10 sequences from bottom branch were used for Multiple sequence alignment (MSA) on https://www.ebi.ac.uk/Tools/msa/ with Maftt method. The alignment result was visulized in Jalview software. The sequence consensus was analyzed at https://weblogo.berkeley.edu. (**Fig. S2**)

Kinetics Measurement of *Nn*SULT1C1

The purified *Nn*SULT1C1 was buffer exchanged to 10 mM NH_4OAc buffer pH 8 via PD-10 column. The concentration of enzyme was calculated based on its Absorption at 280 nm. The enzymatic reaction was performed in 100 μL 10 mM NH_4OAc buffer pH 8 including 1 μM *Nn*SULT1C1, 20 μM PAPS, 5 mM $CaCl_2$ and variable concentration of tyrosine. The reactions were incubated at 37°C and quenched with 100 μL ACN at 5 minutes. The supernatants of these mixtures were used for sTyr quantification via ESI-MS. To improve the sensitivity of sTyr detection, Selected Ion Monitoring (SIM) mode was used to detect the negative ion (m/z=260) under 50 °C drying gas temperature and 2400 V capillary voltage. The standard curve of authentic sTyr was prepared under the same condition, which yields the linear relationship between area under the curve and its sTyr concentration. To obtain the concentration of sTyr produced in enzymatic reaction, its area under the curve was used to calculate sTyr concentration based on the equation obtained from standard curve. Each sample was carried out in n=3 independent samples. The data was fitted to Michaelis-Menten equation in Prism.

Protein Purification from Mammalian Cells

To confirm the genetic incorporation of sTyr from either biosynthesis or external addition, HEK293T and HEK293T-*Nn*SULT1C1 cells were transfected with pAcBac2.tR4-sTyrRS/GFP* with Polyjet In Vitro DNA Transfection Reagent (SignaGen Laboratories) in the presence or absence of 3 mM sTyr addition. Mediums were changed at 12-16 hour after transfection. After 48 hours of transfection, cells were harvested with trypsin and subsequently washed by DPBS for 3 times. Cells were lysed using the Mammalian Cell PE LB reagent (G-Bioscience) according to its manual. The cell lysates were centrifuged at 15,000 rpm for 10 minutes. The protein in the supernatant was purified from the supernatant using Ni-NTA resin (Qiagen) following the manufacturer's instruction. The purified protein was used for ESI-MS analysis.

Mammalian Cell sTyr Concentration Measurement

HEK293T and HEK293T-*Nn*SULT1C1 were detached from plate with trypsin and washed with DPBS for 3 times. The number of cells was counted by hemocytometer. Cells were resuspended in 0.5 mL methanol-water (2:3) and lyzed by six freeze-thaw cycles. The resulting cell lysates were centrifuged at 21000 × g for 1h at 4℃. The resuting supernantants were injected to LC-MS for the quantification of sTyr with selected ion monitoring (SIM) mode. An Agilent 1260 Infinity II LC System coupled with Single Quadrupole ESI-MS System was used for the analysis of all samples. To measure the sTyr ions, ions detected were set to Selected Ion Monitoring (SIM) mode (262 m/z) to detect positive ions of sTyr. Standards of 1 μM, 25 μM, 50 μM, 100 μM, 200 μM, and 400 μM of purchased sTyr (Bachem) dissolved in methanol-water (2:3) were also prepared and analyzed by the same method. A linear standard curve was generated based on peak areas corresponding to sTyr ions and the concentration of sTyr in standard samples. The standard curve was then used to calculate the concentration of

sTyr from different cell lysates. The intracellular concentration of sTyr in cells was calculated with the following equation.

$$[sTyr\ intracellular] = \frac{sTyr\ concentration\ in\ lysate\ \times volumn\ of\ lysate}{total\ cell\ numbers\ \times\ cell\ volumn}$$

2 pL was used as an average volume of mammalian cells.

Mass Spectra Methods For Proteins

A single quadrupole mass spectrometer (Agilent: G7129A) coupled with 1260 infinity II Quaternary Pump (Agilent: G7111B) was used for all the protein samples with PLRP-S (1000A, 5 μm) column. Water with 0.1% formic acid and ACN with 0.1% formic acid were the organic and aqueous mobile phase, respectively. Flow gradient was initially set at 5% ACN, 15% ACN at 0.1 min, 55% ACN at 4.5 min and then back to 10% ACN at 5 min. Spectra were deconvoluted using the Maximum Entropy deconvolution algorithm in the software BioConfirm.
