# Supplementary material for: Unleashing the potential of noncanonical amino acid biosynthesis to create cells with precision tyrosine sulfation
Source: Nat Commun. 2022 Sep 16;13:5434. doi: 10.1038/s41467-022-33111-4 (PMC9481576; doi:10.1038/s41467-022-33111-4)
Supplement: Supplementary file 2 — Reporting Summary [file 41467_2022_33111_MOESM2_ESM.pdf]

nature portfolio

Corresponding author(s): Han Xiao

Last updated by author(s): Aug 26, 2022

Reporting Summary

Nature Portfolio wishes to improve the reproducibility of the work that we publish. This form provides structure for consistency and transparency in reporting. For further information on Nature Portfolio policies, see our Editorial Policies and the Editorial Policy Checklist.

Statistics

For all statistical analyses, confirm that the following items are present in the figure legend, table legend, main text, or Methods section.

n/a	Confirmed	
☐	☒	The exact sample size (*n*) for each experimental group/condition, given as a discrete number and unit of measurement
☐	☒	A statement on whether measurements were taken from distinct samples or whether the same sample was measured repeatedly
☐	☒	The statistical test(s) used AND whether they are one- or two-sided<br>*Only common tests should be described solely by name; describe more complex techniques in the Methods section.*
☒	☐	A description of all covariates tested
☒	☐	A description of any assumptions or corrections, such as tests of normality and adjustment for multiple comparisons
☐	☒	A full description of the statistical parameters including central tendency (e.g. means) or other basic estimates (e.g. regression coefficient) AND variation (e.g. standard deviation) or associated estimates of uncertainty (e.g. confidence intervals)
☐	☒	For null hypothesis testing, the test statistic (e.g. *F*, *t*, *r*) with confidence intervals, effect sizes, degrees of freedom and *P* value noted<br>*Give P values as exact values whenever suitable.*
☒	☐	For Bayesian analysis, information on the choice of priors and Markov chain Monte Carlo settings
☒	☐	For hierarchical and complex designs, identification of the appropriate level for tests and full reporting of outcomes
☒	☐	Estimates of effect sizes (e.g. Cohen's *d*, Pearson's *r*), indicating how they were calculated

Our web collection on statistics for biologists contains articles on many of the points above.

Software and code

Policy information about availability of computer code

Data collection	OpenLAB CDS ChemStation Edition Rev C.01.08[210], Amersham Imager 600, Tecan i-control 3.9.1.0, Zeiss Zen 3.2, Glide v8.1 in Schrödinger software package v2018. AlphaFold v2.1.1
Data analysis	Agilent MassHunter Bioconfirm B.09.00, FlowJo 10.5.3, ImageQuant TL version 8.1, Zeiss Zen 3.1, GraphPad Prism version 6, Pymol 2.1.1, Jalview 2.11.2.0, MEGA 10.2.4, Cytoscape 3.8.2,

For manuscripts utilizing custom algorithms or software that are central to the research but not yet described in published literature, software must be made available to editors and reviewers. We strongly encourage code deposition in a community repository (e.g. GitHub). See the Nature Portfolio guidelines for submitting code & software for further information.

Data

Policy information about availability of data

All manuscripts must include a data availability statement. This statement should provide the following information, where applicable:

- Accession codes, unique identifiers, or web links for publicly available datasets
- A description of any restrictions on data availability
- For clinical datasets or third party data, please ensure that the statement adheres to our policy

All data generated in this study are included in the article and supplementary information. Plasmids for pEvol-NnSULT1C1-cysDNCQ, pET22b-T5-chi28TAG, pET22b-T5-chi31TAG, pET22b-T5-chi28TAG31TAG, pET22b-T5-mad32TAG, pET22b-T5-mad35TAG, pET22b-T5-mad32TAG35TAG, as well as other essential constructs developed by this work, are available on Addgene via https://www.addgene.org/Han_Xiao/. Source data are provided with this paper.

March 2021

Field-specific reporting

Please select the one below that is the best fit for your research. If you are not sure, read the appropriate sections before making your selection.

- [x] Life sciences
- [ ] Behavioural & social sciences
- [ ] Ecological, evolutionary & environmental sciences

For a reference copy of the document with all sections, see nature.com/documents/nr-reporting-summary-flat.pdf

Life sciences study design

All studies must disclose on these points even when the disclosure is negative.

Sample size	Normalized fluorescence measurement were performed in triplicate. Sample size was determined based on a large number of reported studies with a similar methods and purpose.
Data exclusions	No data was excluded.
Replication	All experiments were reproduced two times. Both attempts were successful.
Randomization	Randomization is not relevant to cell-based experiments. Cell number and condition for experiments were well controlled in this study.
Blinding	Investigators were not blinded in the study because there is no clinical studies. There is no bias for the data included in the study.

Reporting for specific materials, systems and methods

We require information from authors about some types of materials, experimental systems and methods used in many studies. Here, indicate whether each material, system or method listed is relevant to your study. If you are not sure if a list item applies to your research, read the appropriate section before selecting a response.

Materials & experimental systems

n/a	Involved in the study	
x		Antibodies
	x	Eukaryotic cell lines
x		Palaeontology and archaeology
x		Animals and other organisms
x		Human research participants
x		Clinical data
x		Dual use research of concern

Methods

n/a	Involved in the study	
x		ChIP-seq
	x	Flow cytometry
x		MRI-based neuroimaging

Eukaryotic cell lines

Policy information about cell lines

Cell line source(s)	HEK293T was purchased from ATCC.
Authentication	HEK293T-NnSULT1C1 was not authenticated. HEK293T is authenticated by the supplier using STR analysis.
Mycoplasma contamination	HEK293T and HEK293T-NnSULT1C1 were regularly monitored for mycoplasma and they are negative.
Commonly misidentified lines (See ICLAC register)	no commonly misidentified cell lines were used.

Flow Cytometry

Plots

Confirm that:

- [x] The axis labels state the marker and fluorochrome used (e.g. CD4-FITC).
- [x] The axis scales are clearly visible. Include numbers along axes only for bottom left plot of group (a 'group' is an analysis of identical markers).
- [x] All plots are contour plots with outliers or pseudocolor plots.
- [x] A numerical value for number of cells or percentage (with statistics) is provided.

Methodology

Sample preparation	HEK293T and HEK293T-NnSULT1C1 cells were transfected with pAcBacw.tR4-sTyrRS/GFP* with Polyjet In Vitro DNA Transfection Reagent (SignaGen Laboratories) in the presence or absence of the indicated concentration of sTyr. Mediums were changed 12-16 hours after transfection. After 48 hours of the transfection, cells were washed with PBS (pH 7.4) and then used for flow cytometry analysis with Sony SA3800 Flow Cytometer where a total of 20,000 cells were analyzed for each sample. Data were processed with FlowJo. Reported data is the average measurement of three samples prepared at the same time with the standard deviation.
Instrument	Sony SA3800 Flow Cytometer
Software	FlowJo 10.5.3
Cell population abundance	A total of 20,000 cells were analyzed for each group.
Gating strategy	The initials "cells" was drawn on FSC/SSC plot. The population of cells with proper FSC and SSC were gated as single cells. Those gated single cells were drawn on a histogram where X axis denotes GFP intensity and y axis denotes cell cumber. The GFP positive population were gated based on blank HEK293T. The gating and analysis strategy were applied to all sample groups.

☒ Tick this box to confirm that a figure exemplifying the gating strategy is provided in the Supplementary Information.
