# Supplementary material for: Unleashing the potential of noncanonical amino acid biosynthesis to create cells with precision tyrosine sulfation
Source: Nat Commun. 2022 Sep 16;13:5434. doi: 10.1038/s41467-022-33111-4 (PMC9481576; doi:10.1038/s41467-022-33111-4)
Supplement: Supplementary file 3 — Description of Additional Supplementary Files [file 41467_2022_33111_MOESM3_ESM.pdf]

Title: Supplementary Data 1:
Description: DNA oligos used in this study.
