# Supplementary material for: Unleashing the potential of noncanonical amino acid biosynthesis to create cells with precision tyrosine sulfation
Source: Nat Commun. 2022 Sep 16;13:5434. doi: 10.1038/s41467-022-33111-4 (PMC9481576; doi:10.1038/s41467-022-33111-4)
Supplement: Supplementary file 4 — Supplementary Data 1 [file 41467_2022_33111_MOESM4_ESM.docx]

| Oligonucleotide | Sequence (5’-3’) | Note |
| --- | --- | --- |
| Da343 | cataaaatcacctcaacctctagatacc |  |
| Da344 | taagtcgaccgatgcccttgag |  |
| Da326 | gaattcattaaagaggagaaattacatATGGAATTGATTCAAGATACGAGCCGCC | HsSULT1A1 |
| Da327 | gctctcaagggcatcggtcgacttaTCACAGCTCACTACGAAAGCTAAGCGAG | HsSULT1A1 |
| Da328 | gaattcattaaagaggagaaattacatATGGAGTTCTCTCGCCCTCCACTTGTGC | HsSULT1A3 |
| Da329 | gctctcaagggcatcggtcgacttaTCACAATTCGCAACGAAACTTGAAATCG | HsSULT1A3 |
| Da330 | gaattcattaaagaggagaaattacatATGGAACTTATCCAGGATACCTCCCGTC | RnSULT1A1 |
| Da331 | gctctcaagggcatcggtcgacttaTCAAAGCTCCGAGCGAAACGACAGTGAAC | RnSULT1A1 |
| Da332 | gaattcattaaagaggagaaattacatATGGCTCTGGACAAGATGGAAAACTTG | GgSULT1C1 |
| Da333 | gctctcaagggcatcggtcgacttaTCAAAGTTCCATACGGAAAACCAAGCTAG | GgSULT1C1 |
| Da345 | gtatctagaggttgaggtgattttATGGCCTTAACATCTGACCTTGGTAAG | O00338 |
| Da346 | gctctcaagggcatcggtcgacttaTCAGAGTTCCATGCAGAAGTTGATGC | O00338 |
| Da347 | gtatctagaggttgaggtgattttATGGCCTTAACTTCAGAGTTAGGGAAAC | A0A1D5RFL7 |
| Da348 | gctctcaagggcatcggtcgacttaTCACAGCTCCATACAGAAGTTAATACTTGTG | A0A1D5RFL7 |
| Da349 | gtatctagaggttgaggtgattttATGGCTCAGGTTCCTGAATTATCGAAACCG | A0A1U7RFW1 |
| Da350 | gctctcaagggcatcggtcgacttaTCACAGTTTCATACAAAAGTTAATCGAAGTGC | A0A1U7RFW1 |
| Da351 | gtatctagaggttgaggtgattttATGCTTTTAATCAGCACATATGCAAAAGCG | A0A1V4J451 |
| Da352 | gctctcaagggcatcggtcgacttaTCATTACAGTTCTGTACGGAAAACAAGTGAAG | A0A1V4J451 |
| Da353 | gtatctagaggttgaggtgattttATGATTGAGCAAAACGGGGACGTG | A0A2I3LMU6 |
| Da354 | gctctcaagggcatcggtcgacttaTCACAGTTCCATACAAAAATTGATCGAGGTC | A0A2I3LMU6 |
| Da355 | gtatctagaggttgaggtgattttATGGCATTAACATCTGAGCTTGGTAAGC | A0A2I3MW57 |
| Da356 | gctctcaagggcatcggtcgacttaTCACAGTTCCATGCAGAAGTTAATACTTGTCC | A0A2I3MW57 |
| Da357 | gtatctagaggttgaggtgattttATGGATATGATTGAGCAGAACGGCG | A0A2I3RUG4 |
| Da358 | gctctcaagggcatcggtcgacttaTCACAGCTCCATGCAGAAATTAATTGAAGTACC | A0A2I3RUG4 |
| Da359 | gtatctagaggttgaggtgattttATGGCGTTAACCTCGGACTTGG | A0A2J8IUC2 |
| Da360 | gctctcaagggcatcggtcgacttaTCACAGTTCCATACAAAAGTTAATAGCCGTCC | A0A2J8IUC2 |
| Da361 | gtatctagaggttgaggtgattttATGGCTCTGACTTCTGAACTGGGGAAAC | A0A2J8R7Z0 |
| Da362 | gctctcaagggcatcggtcgacttaTCACAGCTCCATACAAAAATTAATTGCAGTGC | A0A2J8R7Z0 |
| Da363 | gtatctagaggttgaggtgattttATGGCATTAACAAGCGAGTTGGGGAAG | A0A2K5KXT3 |
| Da364 | gctctcaagggcatcggtcgacttaTCACAGCTCCATGCAAAAGTTAATGCTTGTG | A0A2K5KXT3 |
| Da365 | gtatctagaggttgaggtgattttATGGCTCTCGACAAAATGGAGGAC | A0A091VQH7 |
| Da366 | gctctcaagggcatcggtcgacttaTCACAGCTCAGTGCGAAAGACCAAC | A0A091VQH7 |
| Da367 | gtatctagaggttgaggtgattttATGGCCCTTATCACTGCAGGTACTC | A0A212CXP1 |
| Da368 | gctctcaagggcatcggtcgacttaTCACAGTTCTGTGCAAAAATTGATGCTGGTAC | A0A212CXP1 |
| Da369 | gtatctagaggttgaggtgattttAACATGGAGCTTATCAAAGACATTTCGCG | A0A061I6K0 |
| Da370 | gctctcaagggcatcggtcgacttaTCACAGGTTGCACCGAAATTTGAGG | A0A061I6K0 |
| Da371 | gtatctagaggttgaggtgattttATGGCGCAAAATCCAAGCAACATG | E9QNL5 |
| Da372 | gctctcaagggcatcggtcgacttaTCAAATTTGACAACGAAATGTGAAATCGCAACCG | E9QNL5 |
| Da373 | gtatctagaggttgaggtgattttATGGAACCACTGCGGAAGCC | P52840 |
| Da374 | gctctcaagggcatcggtcgacttaTCAAATTTGGCACCGAAAAGTGAAGTCG | P52840 |
| Da375 | gtatctagaggttgaggtgattttATGGCCCAGAACCCATCTAACATG | Q9R1S5 |
| Da376 | gctctcaagggcatcggtcgacttaTCAAATTTGACACCGGAATGTGAAGTCAC | Q9R1S5 |
| Da377 | gtatctagaggttgaggtgattttATGGCGGGCGAAGATCACAC | G5CB87 |
| Da378 | gctctcaagggcatcggtcgacttaTCAAATTTCAGTGCGGAATGTAAGGGTAC | G5CB87 |
| Da379 | gtatctagaggttgaggtgattttATGCGGAAACCTGAGCTGGAG | G5CB88 |
| Da380 | gctctcaagggcatcggtcgacttaTCACAGTTCGAGGCAAAACCGG | G5CB88 |
| Da381 | gtatctagaggttgaggtgattttATGGCCCTCACTAGCGAACTTG | G7PMW7 |
| Da382 | gctctcaagggcatcggtcgacttaTCACAGTTCCATACAGAAGTTAATGGACGTG | G7PMW7 |
| Da383 | gtatctagaggttgaggtgattttATGGCTTTAACTACCGCGGGTAC | L8IYP9 |
| Da384 | gctctcaagggcatcggtcgacttaTCACAGTTCGGTGCAGAAGTTAATACTTGTC | L8IYP9 |
| Da425 | gtatctagaggttgaggtgattttATGGCTTTGGATAAGATGGAAGACCTCTC | A0A087QVZ4 |
| Da426 | gctctcaagggcatcggtcgacttaTCAGAGTTCTGTGCGGAAGACTACG | A0A087QVZ4 |
| Da427 | gtatctagaggttgaggtgattttATGGCGCTGGACAAAATGAAGGAC | A0A091M6P2 |
| Da428 | gctctcaagggcatcggtcgacttaTCACTCCATACGAAAGACCAGAGATGTG | A0A091M6P2 |
| Da429 | gtatctagaggttgaggtgattttATGCGTATGGAAGATCTTTCGCTGAAATAC | A0A091VNG6 |
| Da430 | gctctcaagggcatcggtcgacttaTCACAATTCCATGCGGAAGACTAACG | A0A091VNG6 |
| Da433 | gtatctagaggttgaggtgattttATGTGCAATGTGTTCCAGATCACTACCG | U3JLS0 |
| Da434 | gctctcaagggcatcggtcgacttaTCACAACTCTGCCCGAAACACC | U3JLS0 |
| Da435 | gtatctagaggttgaggtgattttATGGTAGACAAAATGAAAGACCTCTCACTC | A0A093Q5M0 |
| Da436 | gctctcaagggcatcggtcgacttaTCATAATTCCATGCGGAAAACCAACGAG | A0A093Q5M0 |
| Da437 | gtatctagaggttgaggtgattttATGCTGGCCATGGACAAGATGAAAG | H0ZHC5 |
| Da438 | gctctcaagggcatcggtcgacttaTCACAATTCCATACGGAAGACGAGGC | H0ZHC5 |
| Da439 | gtatctagaggttgaggtgattttATGTCTGGCACTACATGGACTCAGG | A0A091NU80 |
| Da440 | gctctcaagggcatcggtcgacttaTCAAAGTTCCGTCCGGAAAACAAGTG | A0A091NU80 |
| Da443 | gcatgctcgagcagctcag |  |
| Da444 | agatctaattcctcctgttagcccaaaaaaacg |  |
| Da441 | tgggctaacaggaggaattagatctATGGCTCTCGACAAAATGGAGGAC |  |
| Da442 | cgaccctgagctgctcgagcatgcaGAAGACAGTCATAAGTGCGGCGA |  |
| Da445 | atgggattcctcaaagcgtaaacaacgtataac |  |
| Da446 | gtttacgctttgaggaatcccatATGGATCAAATACGACTTACTCACCTGCG |  |
| Da447 | cgaccctgagctgctcgagcatgcTCAGGATCTGATAATATCGTTCTGTCTCAACAG |  |
| Da448 | TCAGGATCTGATAATATCGTTCTGTCTCAACAG |  |
| Da449 | gacagaacgatattatcagatcctgaTAAGTTAACACCGCTCACAGAGACGAG |  |
| Da450 | cgaccctgagctgctcgagcatgcTTAGTAAATAGACACTCTGAACCCCGGATTC |  |
| Da454 | gtcgaccatcatcatcatcatcattgagtttaaac |  |
| Da462 | TCACTATAGGGAGACCCAAGCTGGCTAGCGCCACCATGGCGAGTTCCAATCTGATTAAGC |  |
| Da463 | GAGTTAAAGTCGACTTAACGCGTTGAATTCTTATACAGGTCCTTTCCAGCAAATGAGAC |  |
| Da556 | attcattaaagaggagaaattacatATGCAGCCTAAGGAAAAAACAAAAGGTGTAG |  |
| Da557 | gagtccaagctcagctaattaagcttATGAGATTCAGAACTGAAGTCAGGAATCTGATC |  |
| Da558 | attcattaaagaggagaaattacatATGTATCCAGAACGGGACTCCG |  |
| Da559 | gagtccaagctcagctaattaagcttCGGTTTGTTGCCCCGAAGG |  |
| Da584 | TTACGACGAGtagGAGGACAACGC |  |
| Da585 | CTAACGTCCATCTGACGGGCG |  |
| Da586 | GGACGCGGATtagGATGAATATGAGGAAG |  |
| Da587 | CCGTCGGTACGTTTCTGCACC |  |
| Da655 | GGCAAAGAATTGCAAGTTTGTACAAAAAAGCAGGCTGCCACCATGGCGCTTGACAAAATGGAAGAC |  |
| Da656 | GCCTGCACCTGAGGATCACCACTTTGTACAAGAAAGCTGGGTTTAaagctctgtccgaaagacgagagaag |  |
| Da661 | TCCGCGTCCCCGTCGGTA |  |
| Da662 | TTACGATGAAtagGAGGAAGACGGGACGAC |  |
| Da663 | TTAGGATGAAtagGAGGAAGACGGGACGAC |  |
| Da664 | ATCTGACGGGCGGAAATGAGCG |  |
| Da665 | GGACGTTAGTtagGACGAGTACGAGGACAACG |  |
| Da666 | GGACGTTAGTtagGACGAGTAGGAGGACAACG |  |
| Da687 | attcattaaagaggagaaattacatATGGCTCTCGACAAAATGGAGGAC |  |
| Da688 | gagtccaagctcagctaattaagctTTAGTGGTGGTGGTGGTGGTGCAGCTCAGTGCGAAAGACCAAC |  |
| Da858 | CGGCACCCGTTCCTCGAATGGTCTGGGCTTGAATTAGCGGAGGC |  |
| Da859 | CCATTCGAGGAACGGGTGCC |  |
| Da871 | GAAGTCGAAGGTATCCCGTTCGCGAAGCCTATTTGTAGTACGTGGGATCAAGTG |  |
| Da872 | GAACGGGATACCTTCGACTTCGCAG |  |
| Da873 | GAAGGTATCCCGTTCACTAAGCCTGCGTGTAGTACGTGGGATCAAGTGTGGAAATTC |  |
| Da874 | AGGCTTAGTGAACGGGATACCTTCG |  |
| Da875 | GCCGGCACCCGTTCCTCGAAGCGTCAATCCAGGAGCCACCGGCT |  |
| Da876 | TTCGAGGAACGGGTGCCGGC |  |
| Da877 | CTATCACTTTCACCGCATGAGCAAAGCGATGCCAGATCCTGGGACCTGG |  |
| Da878 | TTTGCTCATGCGGTGAAAGTGATAGTAAC |  |
